# An Essential Viral Transcription Activator Modulates Chromatin Dynamics

**DOI:** 10.1371/journal.ppat.1005842

**Published:** 2016-08-30

**Authors:** Rebecca L. Gibeault, Kristen L. Conn, Michael D. Bildersheim, Luis M. Schang

**Affiliations:** 1 Department of Biochemistry, University of Alberta, Edmonton, Alberta, Canada; 2 Department of Medical Microbiology and Immunology, University of Alberta, Edmonton, Alberta, Canada; University of Pennsylvania Medical School, UNITED STATES

## Abstract

Although ICP4 is the only essential transcription activator of herpes simplex virus 1 (HSV-1), its mechanisms of action are still only partially understood. We and others propose a model in which HSV-1 genomes are chromatinized as a cellular defense to inhibit HSV-1 transcription. To counteract silencing, HSV-1 would have evolved proteins that prevent or destabilize chromatinization to activate transcription. These proteins should act as HSV-1 transcription activators. We have shown that HSV-1 genomes are organized in highly dynamic nucleosomes and that histone dynamics increase in cells infected with wild type HSV-1. We now show that whereas HSV-1 mutants encoding no functional ICP0 or VP16 partially enhanced histone dynamics, mutants encoding no functional ICP4 did so only minimally. Transient expression of ICP4 was sufficient to enhance histone dynamics in the absence of other HSV-1 proteins or HSV-1 DNA. The dynamics of H3.1 were increased in cells expressing ICP4 to a greater extent than those of H3.3. The dynamics of H2B were increased in cells expressing ICP4, whereas those of canonical H2A were not. ICP4 preferentially targets silencing H3.1 and may also target the silencing H2A variants. In infected cells, histone dynamics were increased in the viral replication compartments, where ICP4 localizes. These results suggest a mechanism whereby ICP4 activates transcription by disrupting, or preventing the formation of, stable silencing nucleosomes on HSV-1 genomes.

## Author Summary

The nuclear-replicating DNA viruses of the family herpesviridae cause a variety of diseases. Eight herpesviruses infect humans. Three of them, including herpes simplex virus 1 (HSV-1), belong to the alpha-herpesvirus sub-family. Viruses in this family have the fastest replication cycles of all herpesviruses, producing acute symptoms. During lytic infection, the genomes of HSV-1 associate with histones in more dynamic chromatin than those of the beta- and gamma- herpesviruses. The transcription activator ICP4 is conserved only among alpha-herpesviruses. Although ICP4 is essential, relatively little is known about its mechanisms of action. We have shown that histone dynamics are enhanced in HSV-1 lytically infected cells. Here we show that HSV-1 mutants in ICP4 are deficient in their ability to enhance histone dynamics. ICP4 was sufficient to enhance histone dynamics in the absence of other HSV-1 proteins or DNA. The dynamics of histones were greater in the viral replication compartments, where ICP4 localizes, than in the cellular chromatin. ICP4 may thus mobilize histones away from HSV-1 genomes to activate transcription. Such a mechanism of transcription activation would result in the highly dynamic nature of the viral chromatin and the fast replication cycles, and the acute pathologies, of the alpha-herpesviruses.

## Introduction

The genes of the nuclear-replicating double stranded (ds) DNA virus herpes simplex virus 1 (HSV-1) are expressed in a coordinate manner. VP16, a virion protein, first activates expression of the five immediate early (IE) genes, in part through the recruitment of the histone demethylase LSD1 and histone acetyltransferase CBP/p300 to IE promoters [1–5]. Two IE proteins, ICPO and ICP4, then activate transcription of the early (E) genes, which encode proteins required for HSV-1 DNA replication and several other functions [6]. Late (L) genes are transcribed after DNA replication. Both ICP0 and ICP4 also contribute to the activation of L gene expression.

The mechanisms whereby VP16 activates IE gene transcription are well characterized [1, 3, 5, 7–12]. In contrast, the mechanisms whereby ICP0 and ICP4 then activate transcription of E and L genes remain only partially understood. ICP4 binds to specific DNA sequences to inhibit transcription of IE genes [13]. However, it does not bind to any specific sequences to activate transcription of E or L genes [14]. Over 141 proteins that interact with ICP4 at 6 h post infection (hpi) were identified by mass spectrometry analyses, including the chromatin remodeling complexes SWI/SNF, Ino80, and NuRD [15]. The histone acetyltransferase CLOCK was identified as another ICP4 interactor by coimmunoprecipitation [16]. ICP4 also interacts with many components of the mediator complex and may activate transcription by a gene looping mechanism [15], promoting the recycling of RNA polymerase II from the 3’ end of a gene back to the transcription start sites.

Whereas HSV-1 genomes are regularly chromatinized in latent infection, HSV-1 genomes are in particularly dynamic chromatin in lytic infections [17]. The basic unit of chromatin is the nucleosome, which consists of two dimers of each of the core histones H2A-H2B and H3-H4 wrapped by 146 base pairs of double stranded DNA. Linker histone H1 further binds DNA at the entry and exit sites of the core nucleosome. Chromatin is dynamic, nucleosomes disassemble and then the released histones diffuse through the nucleus bound to chaperones and re-assemble nucleosomes at different sites. Linker histones are more dynamic than core histones, with their exchanges occurring in minutes or hours, respectively [18–20].

The dynamics of cellular nucleosomes are altered through post-translational modifications to histones and the incorporation of histone variants instead of the canonical ones, among other factors [21–30]. Acetylation of histone tails by histone acetyltransferases generally destabilizes nucleosomes, whereas their methylation by histone methyltransferases destabilizes or stabilizes nucleosomes, depending on the site and the degree of methylation [21–29]. Nucleosomes containing H3.3 are more dynamic than those containing H3.1 [30]. Canonical histone H3.1 is assembled in chromatin with newly synthesized DNA by the histone chaperone CAF-1, whereas variant H3.3, which differs by only 5 amino acid residues, is assembled in the chromatin of transcribed genes or telomeres by HIRA or DAXX, respectively, independently of DNA replication [31–36]. H3.3 is typically post-translationally modified with more markers of active transcription than H3.1, such as K4 and K79 methylation and K9, K14 and K23 acetylation [37].

We had found that histone dynamics increase during infection with wild type HSV-1 [38–40]. Histone dynamics still increased in infected cells treated with phosphonoacetic acid, indicating that neither HSV-1 DNA replication nor L gene expression are required, whereas they were largely unaffected by UV-inactivated HSV-1, indicating that virion attachment or entry are not sufficient. Therefore, IE or E proteins most likely affect histone dynamics.

We and others propose a model in which the chromatinization of HSV-1 DNA is a cellular defense mechanism to silence HSV-1 gene expression. To counteract this mechanism, HSV-1 would have evolved proteins that prevent or disrupt the stable chromatinization of HSV-1 genomes. This nucleosome destabilization process would increase histone dynamics and promote transcription. Under this model, one or more of the three HSV-1 transcription activators would be expected to enhance histone dynamics.

Here we report that HSV-1 mutants encoding no functional VP16, ICP0 or ICP4 still enhance histone dynamics, but to a much lesser extent than wild type HSV-1. We further show that an HSV-1 mutant encoding no functional ICP4 is the most deficient in enhancing histone dynamics. Transient expression of ICP4 was sufficient to enhance histone dynamics in the absence of any other HSV-1 protein or DNA. ICP4 may moreover preferentially target silencing histone variants, such as H3.1. The dynamics of canonical H2A were not enhanced in cells expressing ICP4, suggesting that other H2A variants may be targeted by ICP4. During lytic infections, histones were more dynamic in the replication compartments, where ICP4 localizes, than in the cellular chromatin. Together, our results suggest a novel mechanism of transcription activation by ICP4, in which ICP4 prevents the formation of stable nucleosomes on HSV-1 genomes, or destabilizes preformed ones, to promote transcription by allowing access of the RNA polymerase II complex to the HSV-1 genes.

## Results

### Functional ICP4 or E proteins are required to enhance histone dynamics beyond a basal level

IE or E proteins enhance linker and core histone dynamics during HSV-1 infection [38–40]. To test whether the enhanced dynamics required the expression of ICP4 or any E protein, we used HSV-1 strain n12, which expresses a transactivation incompetent truncated ICP4 [41]. Consequently, IE proteins other than ICP4 are expressed to high levels in the absence of any E or L protein expression or DNA replication. The levels of green-fluorescent protein (GFP)-histone fusion proteins in the free pools, and the initial rates of fluorescence recovery after photobleaching (core histones), or time to recover 50% of the relative fluorescence in the photobleached region (T_50_; for linker histone H1.2), were evaluated to analyze histone dynamics [38–40]. The fluorescence recovery of histones is biphasic [18, 20, 42]. The initial, faster, phase of fluorescence recovery, analyzed by the slope of the fluorescence recovery between the first two times, reflects histones assembled in the most dynamic chromatin, such as those in rapidly transcribed genes. The later, slower, phase of fluorescence recovery, analyzed by the slope of the fluorescence recovery between 25 and 100 seconds for core histones, reflects the histones assembled in less dynamic chromatin. The relative fluorescence intensity immediately after photobleaching reflects the “free pool” of histones, as only histones not in chromatin diffuse in and out the bleached region during photobleaching. The global dynamics of linker histones are described by the T_50_, which is the most sensitive parameter.

**Fig 1:**
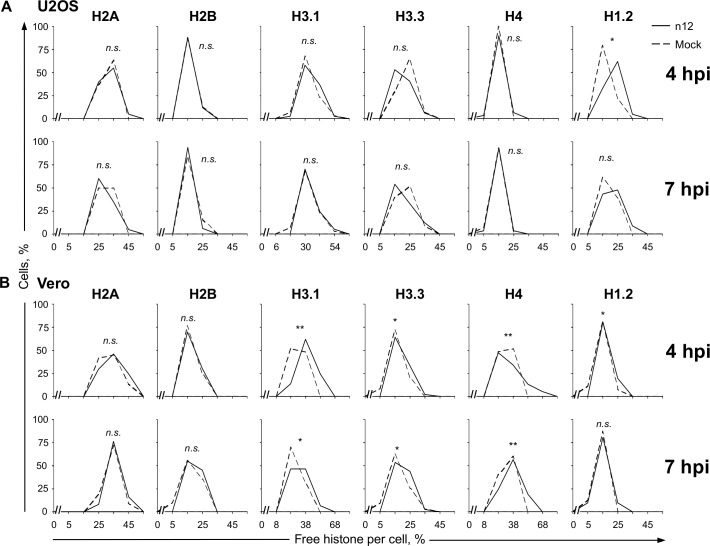
The dynamics of linker and core histones are only minimally altered in the absence of functional ICP4. U2OS (**A**) or Vero (**B**) cells were transfected with plasmids expressing GFP fused to H2A, H2B, H3.1, H3.3, H4, or H1.2. Transfected cells were mock infected or infected with 30 plaque forming units (PFU) per cell of HSV-1 strain n12 and histone dynamics were examined from 4 to 5 or 7 to 8 hours post infection (hpi) (**4 hpi or 7 hpi**, respectively) by FRAP. Frequency distribution plots showing the percentage of free GFP-H2A, -H2B, -H3.1, -H3.3, -H4, or -H1.2 per individual mock- (dashed line) or n12 (solid line) infected cell at 4 or 7 hpi. **, P < 0.01; *, P < 0.05; *n*.*s*., not significant. n ≥ 20 cells from at least 3 independent experiments, except for U2OS H2A (n = 20 cells from 2 independent experiments).

The levels of all free histones had unimodal normal frequency distributions throughout the population of n12 infected U2OS cells (Fig 1A). n12 infection of U2OS cells was not sufficient to increase the free pools of any core histone, whereas those of H1.2 were only increased to a basal degree at early times after infection (Fig 1A; P<0.05). The levels of all free histones also had unimodal normal frequency distributions throughout the population of n12 infected Vero cells (Fig 1B). The free pools of H3.1, H3.3, and H4 were also increased at 4 and 7 hpi in n12 infected Vero cells, although less than in KOS infected cells [39, 40].

**Fig 2:**
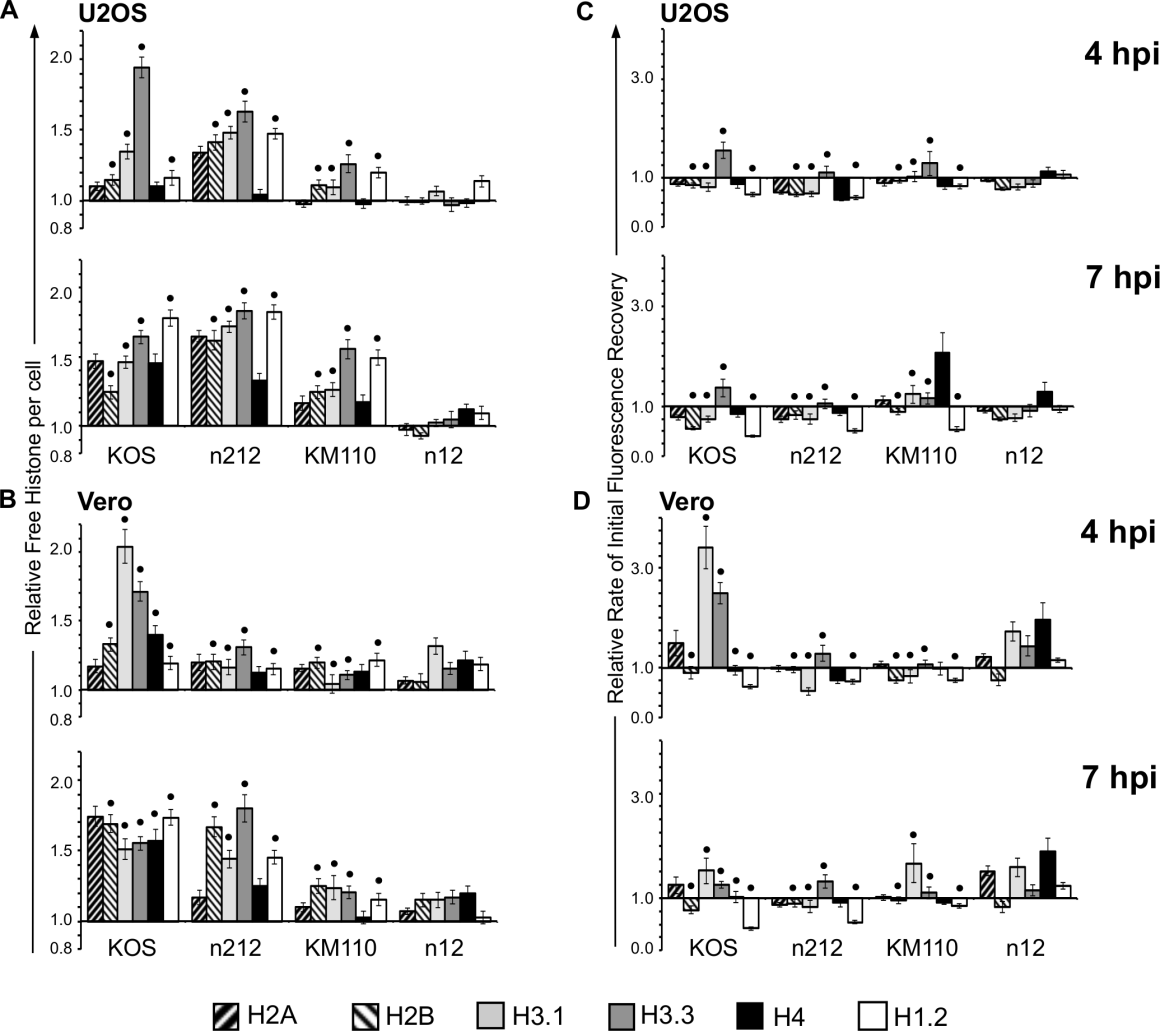
Core and linker histone dynamics during infection with wild-type or mutant HSV-1 strains defective in VP16, ICP0 or ICP4. U2OS or Vero cells were transfected with plasmids expressing GFP fused to H2A, H2B, H3.1, H3.3, H4, or H1.2. Transfected cells were mock-infected or infected with 30 PFU per cell of HSV-1 strain KOS, n212, KM110, or n12, or 6 PFU per cell of strain KOS (U2OS cells). Histone dynamics were evaluated from 4 to 5 (**4 hpi**) or 7 to 8 (**7 hpi**) hpi by FRAP. A), B) Bar graphs showing the average levels of free GFP-H2A, -H2B, -H3.1, -H3.3, -H4, or -H1.2 relative to those in mock-infected cells (set at 1.0) at 4 or 7 hpi. C), D) Bar graphs showing the average initial rates of normalized fluorescence recovery (core histones) or the average T_50_ (H1.2) relative to those in mock-infected cells (set at 1.0) at 4 or 7 hpi. Error bars, SEM. (**•**) Data for GFP-H2B, -H3.1, -H3.3, and -H1.2 for HSV-1 strains KOS, n212, and KM110 (Vero and U2OS) and GFP-H4 for HSV-1 strain KOS (Vero), included for comparison, are from previously published experiments [38–40].

We had previously shown that the enhancement of histone dynamics in Vero cells infected with an HSV-1 mutant in ICP0 was partly impaired, such that the enhanced late increase of histone dynamics ultimately occurs (Fig 2A and 2B, n212 [38–40]). The pools of some free histones were increased to an even larger degree at 7 hpi in the absence of ICP0 (Fig 2A, n212 [38–40]). The double ICP0 and VP16 HSV-1 mutant, which expresses little ICP4 in Vero cells [43], enhanced histone dynamics to almost only a basal level in these cells (Fig 2A and 2B, KM110 [38–40]). Vero cells infected with ICP4 mutant n12 only had a basal increase in the levels of free linker and core histones, which was not further enhanced at later times after infection (Figs 1B and 2). Expression of ICP0, ICP22, ICP27, or ICP47 in the absence of functional ICP4 (and E proteins) is thus not sufficient to increase the pools of free core or linker histones to the same degree as infection with wild-type or ICP0 or VP16 mutant strains of HSV-1 (Fig 2 [38–40]).

**Fig 3:**
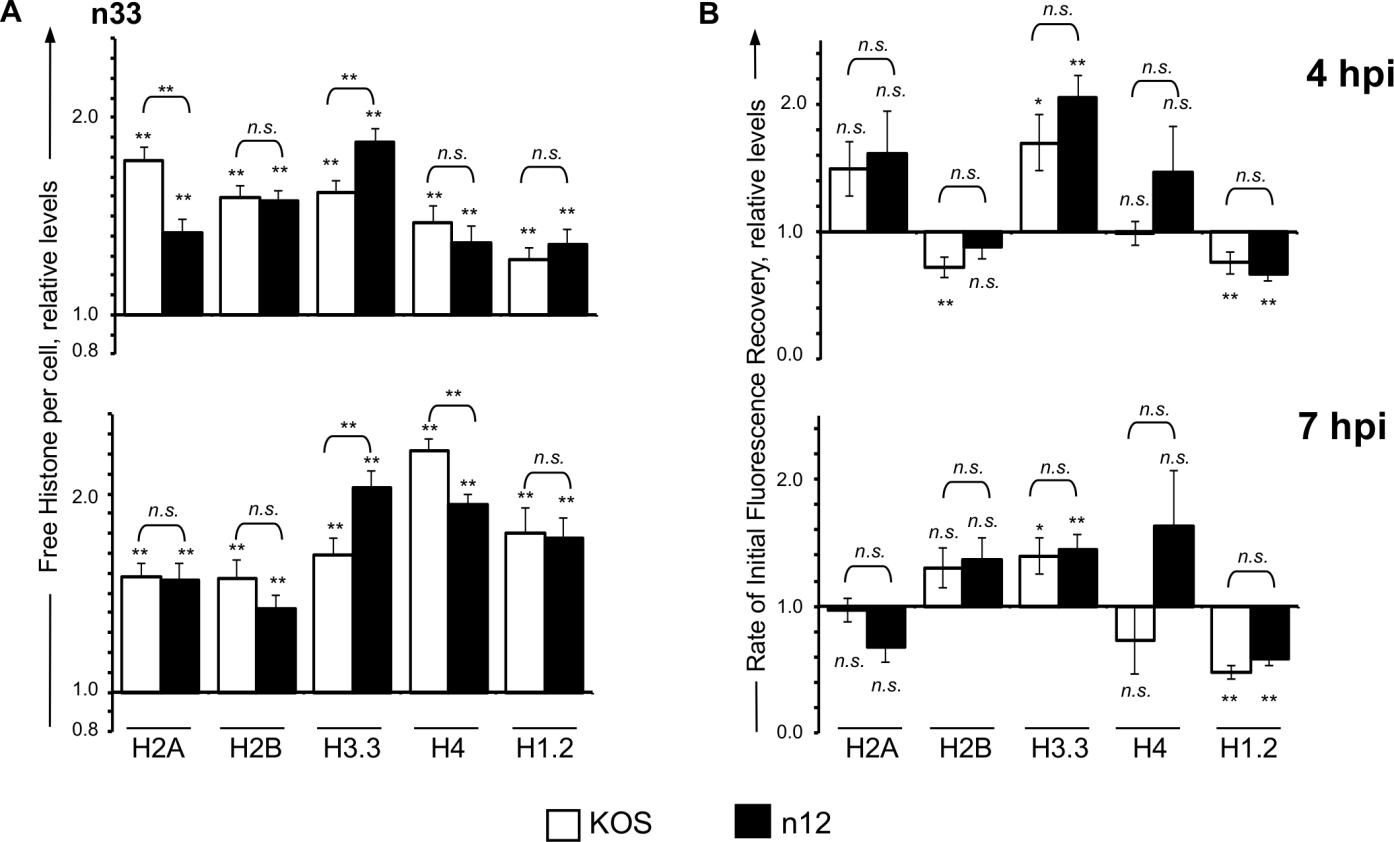
Functional ICP4 enhances histone dynamics during n12 infection. n-33 cells transfected with plasmids expressing GFP fused to H2A, H2B, H3.3, H4, or H1.2 were mock infected or infected with 30 PFU per cell of HSV-1 strain KOS (☐) or n12 (■). Histone dynamics were evaluated from 4 to 5 (**4 hpi**) or 7 to 8 (**7 hpi**) hpi by FRAP. A) Bar graphs showing the average levels of free GFP-H2A, -H2B, -H3.3, -H4, or -H1.2 in KOS- or n12- infected cells relative to those in mock-infected cells (set at 1.0) at 4 or 7 hpi. B) Bar graphs showing the average initial rates of normalized fluorescence recovery (core histones) or the average T_50_ (H1.2) in KOS- or n12- infected cells relative to those in mock-infected cells (set at 1.0) at 4 or 7 hpi. Error bars, SEM. **, P < 0.01; *, P < 0.05; *n*.*s*., not significant. n ≥ 15 cells from at least 2 independent experiments, except GFP-H2A and -H4 n ≥ 8 cells from 1 experiment.

To test whether the inability of n12 to enhance histone dynamics above the basal degree was due to unknown mutations within this strain, histone dynamics were re-evaluated in a complementary Vero-derived cell line (n-33) that expresses HSV-2 ICP4 upon infection [44]. The dynamics of core and linker histones were enhanced to approximately the same degree in n-33 cells infected with n12 or wild-type KOS (Fig 3).

### The dynamics of core histones H2B and H4 increase in cells transiently expressing ICP4

Histones are thus minimally mobilized in U2OS or Vero cells infected with an HSV-1 mutant encoding no functional ICP4 (Figs 1–3). ICP4 may induce the increase in histone dynamics by itself. Alternatively, the protein product of an E gene may increase histone dynamics (DNA replication or L proteins are not required [39, 40]), as the expression of E genes requires ICP4. To test these possibilities, we analyzed the effects of ectopically expressed ICP4 in histone dynamics.

H4 and H2B have no major variants, and they therefore represent the entire population of H3-H4 and H2A-H2B dimers, respectively. To evaluate the dynamics of H4 and H2B in cells transiently expressing ICP4, we optimized the co-transfection of GFP-H2B or GFP-H4 with free red fluorescent protein (RFP) or RFP-ICP4 such that approximately half of the cells expressing detectable levels of GFP also expressed detectable levels of the RFP fusion proteins (or free RFP). This approach allows us to analyze histone dynamics in cells expressing detectable or undetectable levels of the test proteins in otherwise identical conditions. GFP fluorescence within the bleached region was normalized to total nuclear fluorescence to account for differences in GFP expression. The relative fluorescence within the bleached region at each time was then normalized to the initial relative fluorescence within the same region prior to photobleaching. The results are therefore independent of the GFP-histone expression levels [38–40].

**Fig 4:**
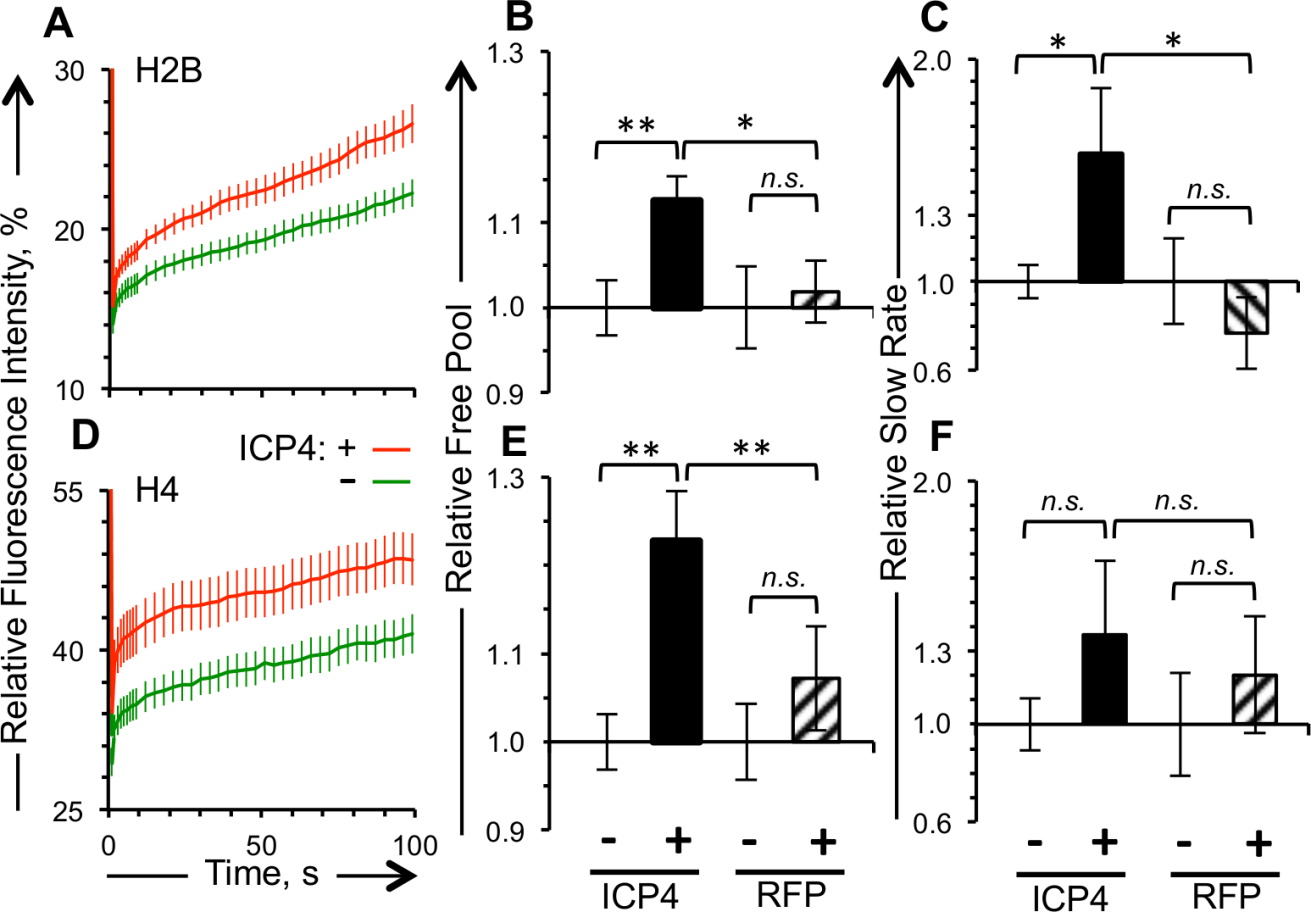
The dynamics of H2B and H4, representative of each histone dimer, are enhanced in cells transiently expressing ICP4. Vero cells were co-transfected with plasmids expressing GFP-H2B or -H4 and RFP-ICP4 or RFP, such that approximately half of the cells expressing detectable levels of GFP-histone also express detectable levels of RFP. A), D) Average fluorescence recovery curves for GFP-H2B and -H4, respectively, for cells expressing detectable (red line) or undetectable (green line) levels of RFP-ICP4. B), E) Bar graphs showing the average levels of free GFP-H2B or -H4, respectively, in cells expressing detectable levels of RFP-ICP4 or RFP relative to those in cells expressing undetectable levels of RFP-ICP4 or RFP, respectively. C), F) Bar graphs showing average GFP-H2B or -H4 slow exchange rate in cells expressing detectable levels of RFP-ICP4 or RFP relative to those in cells expressing undetectable levels of RFP-ICP4 or RFP. Error bars, SEM. **, P < 0.01; *, P < 0.05; *n*.*s*., not significant. n ≥ 15 cells from at least 3 independent experiments.

The free pools of GFP-H4 or -H2B were 22 or 12% greater, respectively, in cells expressing detectable than undetectable levels of RFP-ICP4 (p<0.01) (Fig 4A, 4B, 4D and 4E). As expected, the free pools of GFP-H4 or -H2B were not significantly higher in cells expressing detectable than undetectable levels of free RFP (Fig 4B and 4E). The slow exchange rate of GFP-H2B, which evaluates the dynamics of the H2B molecules in low turnover nucleosomes, was 57% greater in cells expressing detectable than undetectable levels of RFP-ICP4 (p<0.05) (Fig 4C). While the slow exchange rate of GFP-H4 tended to be faster in cells expressing detectable than undetectable levels of RFP-ICP4, it was not significantly so (Fig 4F). The slow exchange rates of GFP-H2B or -H4 were not significantly changed in cells expressing detectable levels of free RFP (Fig 4C and 4F).

### The dynamics of canonical H3.1 and variant H3.3 increase in cells transiently expressing ICP4

H3.3 is initially detected in the nucleosomes assembled with HSV-1 genomes, whereas H3.1 is detected in HSV-1 nucleosomes only after the onset of HSV-1 DNA replication [45]. Consistently, the dynamics of H3.1 and H3.3 are differentially affected in cells infected with wild type HSV-1 [40]. Their free pools decrease between 4 and 7 hpi in Vero cells, but that of H3.1 decreases to a much greater extent (Fig 2). The free pools of H3.1 also decrease between 4 and 7 hpi in U2OS cells, whereas those of H3.3 do not (Fig 2). Whereas the free pool of H3.3 at 7 hpi is not affected by HSV-1 DNA replication, moreover, that of H3.1 is two-fold greater when HSV-1 DNA replication is inhibited [40]. The dynamics of H4 were enhanced in cells transiently expressing ICP4 (Fig 4D and 4E). We therefore expected the dynamics of H3.1 or H3.3, which form dimers with H4, to also be enhanced.

**Fig 5:**
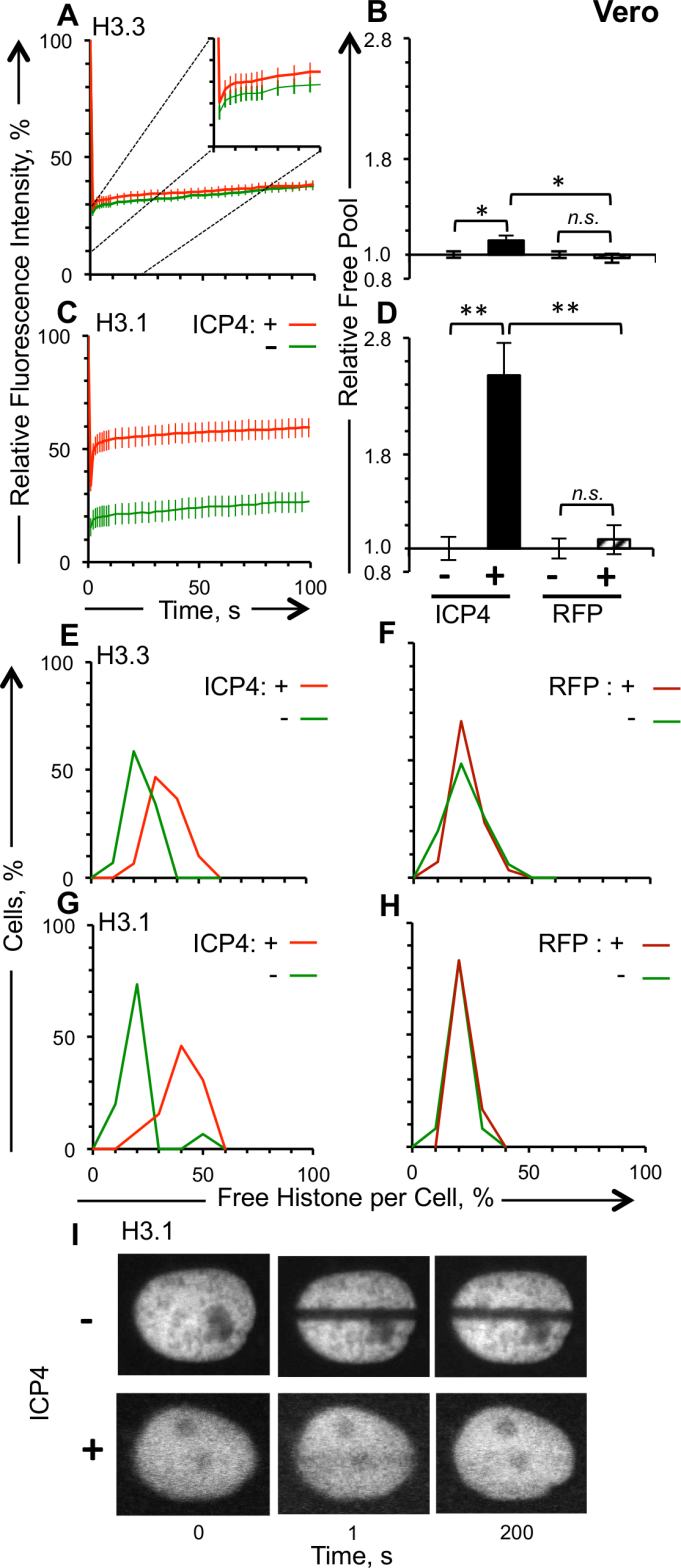
H3.1 dynamics are enhanced more than those of H3.3 in Vero cells transiently expressing ICP4. Vero cells were co-transfected with plasmids expressing GFP-H3.1 or -H3.3 and RFP-ICP4 or RFP, such that approximately half of the cells expressing detectable levels of GFP also express detectable levels of RFP. A), C) Average fluorescence recovery curves for GFP-H3.3 and -H3.1, respectively, for cells expressing detectable (red line) or undetectable (green line) levels of RFP-ICP4. B), D) Bar graphs showing the average levels of free GFP-H3.3 or -H3.1, respectively, in cells expressing detectable levels of RFP-ICP4 or RFP relative to cells expressing undetectable levels of RFP-ICP4 or RFP, respectively. E) Frequency distribution of the free pool of GFP-H3.3 in cells expressing detectable (red line) or undetectable (green line) levels of RFP-ICP4. F) Frequency distribution of the free pool of GFP-H3.3 in cells expressing detectable (dark red line) or undetectable (green line) levels of RFP. G) Frequency distribution of the free pool of H3.1 in cells expressing detectable (red line) or undetectable (green line) levels of RFP-ICP4. H) Frequency distribution of the free pool of GFP-H3.1 in cells expressing detectable (dark red line) or undetectable (green line) levels of RFP. I) Representative images of fluorescent nuclei expressing GFP-H3.1 and detectable or undetectable levels of RFP-ICP4, immediately prior to (T = 0) or after (T = 1) photobleaching, or 200 seconds later. Error bars, SEM. **, P < 0.01; *, P < 0.05; *n*.*s*., not significant. n ≥ 15 cells from at least 3 independent experiments.

The free pool of GFP-H3.3 was 14% greater in Vero cells expressing detectable than undetectable levels of RFP-ICP4 (p<0.05) (Fig 5A and 5B). The unimodal frequency distribution of the GFP-H3.3 free pools had its peak shifted to the right, indicating a larger free pool, in cells expressing detectable ICP4 compared to cells expressing undetectable ICP4 (Fig 5E). In contrast, the frequency distribution of the GFP-H3.3 free pools was not altered in cells expressing detectable or undetectable RFP (Fig 5B and 5F).

GFP-H3.1 was mobilized to a much greater extent (Fig 5C, 5D and 5G). The average free pool of GFP-H3.1 was 248% greater in cells expressing detectable than undetectable levels of RFP-ICP4 (Fig 5D). The frequency distribution curves of the GFP-H3.1 free pools showed moreover that the cells expressing undetectable levels of ICP4 had free pools distributed normally around 20%, whereas the cells expressing detectable ICP4 had a skewed distribution peaking at twice as large (Fig 5G). Cells expressing detectable RFP or not had equally distributed free pools (Fig 5H). The increased dynamics of GFP-H3.1 were also reflected by its nuclear distribution (Fig 5I). GFP-H3.1 had the punctuated localization characteristic of chromatin in cells expressing undetectable levels of RFP-ICP4. In contrast, GFP-H3.1 was diffusely distributed through the nucleus in cells expressing detectable levels of RFP-ICP4, distribution which is consistent with soluble proteins (i.e., free H3.1). The free pools of GFP-H3.1 or -H3.3 were not affected in cells expressing detectable levels of free RFP (Fig 5).

**Fig 6:**
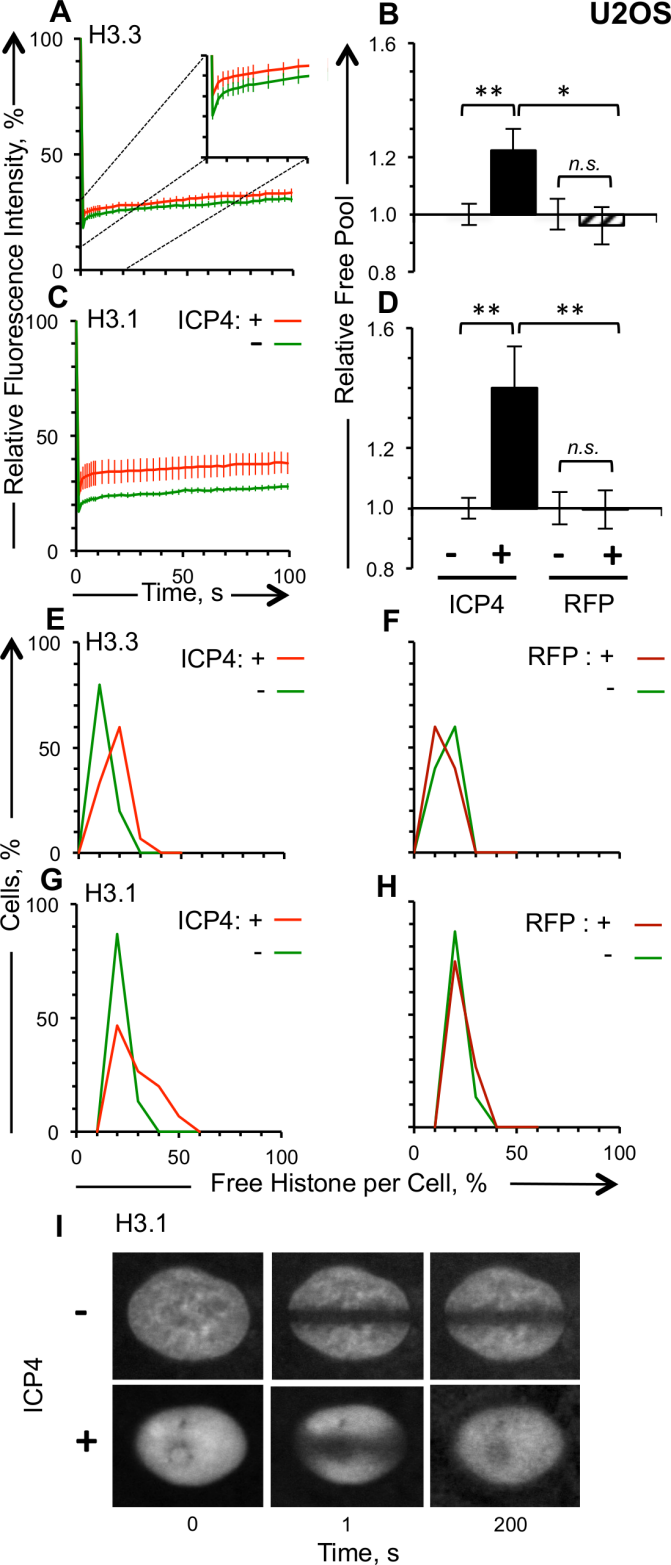
H3.1 dynamics are enhanced more than those of H3.3 in U2OS cells transiently expressing ICP4. U2OS cells were co-transfected with plasmids expressing GFP-H3.1 or -H3.3 and RFP-ICP4 or RFP such that approximately half of the cells expressing detectable levels of GFP also express detectable levels of RFP. A), C) Average fluorescence recovery curves for GFP-H3.3 and -H3.1, respectively, for cells expressing detectable (red line) or undetectable (green line) levels of RFP-ICP4. B), D) Bar graphs showing the average levels of free GFP-H3.3 or -H3.1, respectively, in cells expressing detectable levels of RFP-ICP4 or RFP relative to those in cells expressing undetectable levels of RFP-ICP4 or RFP. E) Frequency distribution of the free pool of GFP-H3.3 in cells expressing detectable (red line) or undetectable (green line) levels of RFP-ICP4. F) Frequency distribution curve of the free pool of GFP-H3.3 in cells expressing detectable (dark red line) or undetectable (green line) levels of RFP. G) Frequency distribution of the free pool of GFP-H3.1 in cells expressing detectable (red line) or undetectable (green line) levels of RFP-ICP4. H) Frequency distribution curve of the free pool of GFP-H3.1 in cells expressing detectable (dark red line) or undetectable (green line) levels of RFP. I) Representative images of fluorescent nuclei expressing GFP-H3.1 and detectable or undetectable levels of RFP-ICP4, immediately prior to (T = 0) or 1 second after (T = 1) photobleaching, or 200 seconds later. Error bars, SEM. **, P < 0.01; *, P < 0.05; *n*.*s*., not significant. n ≥ 15 cells from at least 3 independent experiments.

The free pools of GFP-H3.3 or -H3.1 were also 22% or 40% greater, respectively, in U2OS cells expressing detectable than undetectable levels of RFP-ICP4 (p<0.01) (Fig 6A–6D). Cells expressing undetectable levels of RFP-ICP4 or RFP had equally normally distributed free pools of GFP-H3.1 or H3.3 (Fig 6E, 6F, 6G and 6H). In contrast, cells expressing detectable levels of ICP4 had free pools of GFP-H3.1 with a skewed distribution with a clear shoulder peaking at 40%. The increased dynamics of GFP-H3.1 were also reflected by its nuclear distribution in U2OS cells (Fig 6I).

### The dynamics of canonical H2A were not affected in cells transiently expressing ICP4

**Fig 7:**
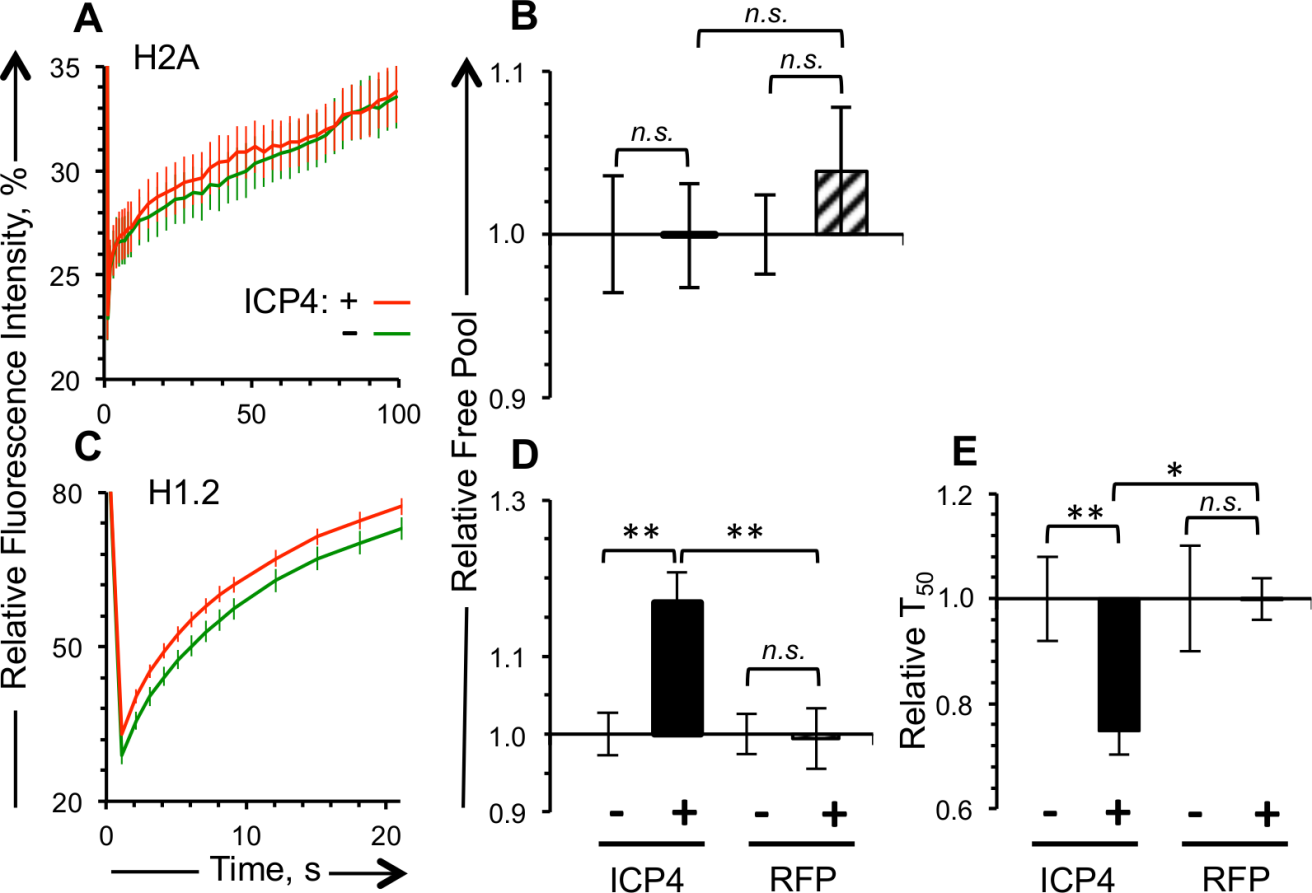
The dynamics of H1.2, but not those of canonical H2A, are enhanced in cells transiently expressing ICP4. Vero cells were co-transfected with plasmids expressing GFP-H2A or -H1.2 and RFP-ICP4 or RFP such that approximately half of the cells expressing detectable levels of GFP also express detectable levels of RFP. A), C) Average fluorescence recovery curves for GFP-H2A and -H1.2, respectively, for cells expressing detectable (red line) or undetectable (green line) levels of RFP-ICP4. B), D) Bar graphs showing the average levels of free GFP-H2A or -H1.2, respectively, in cells expressing detectable levels of RFP-ICP4 or RFP relative to those in cells expressing undetectable levels of RFP-ICP4 or RFP, respectively. E) Bar graphs showing the average T_50_ of GFP-H1.2 in cells expressing detectable levels of RFP-ICP4 or RFP relative to those in cells expressing undetectable levels of RFP-ICP4 or RFP. Error bars, SEM. **, P < 0.01; *, P < 0.05; *n*.*s*., not significant. n ≥ 15 cells from at least 3 independent experiments.

H2B was mobilized in cells expressing ICP4, albeit its free pool increased the least of all core histones (Fig 4). H2B forms dimers with canonical H2A or any of its multiple variants. No H2A variant has been shown to interact with HSV-1 genomes, whereas canonical H2A has. We thus co-transfected cells with plasmids expressing GFP-H2A and RFP-ICP4. Surprisingly, the dynamics of canonical GFP-H2A were not significantly affected in cells expressing detectable levels of RFP-ICP4 (or free RFP) (Fig 7A and 7B).

### The dynamics of linker histone H1.2 were increased in cells transiently expressing ICP4

All linker histones are mobilized in cells infected with wild type HSV-1 [38]. Variant H1.2 was mobilized the most, with a T_50_ in infected cells 60% of that in mock infected cells. H1.2 is synthesized independently of the cell cycle stage and in all cell types that HSV-1 infects. We therefore focused on the mobilization of H1.2 in cells expressing RFP-ICP4.

The T_50_ of GFP-H1.2 in cells expressing detectable levels of RFP-ICP4 was 76% of that in cells expressing undetectable levels of RFP-ICP4 (p<0.01) (Fig 7C and 7E), and the free pools were 17% greater (p<0.01) (Fig 7D). As expected, GFP-H1.2 T_50_ or its free pools were not significantly different in cells expressing detectable or undetectable levels of free RFP (Fig 7D and 7E).

### The truncated, transcriptionally inactive ICP4 n12 mutant does not enhance histone dynamics

**Fig 8:**
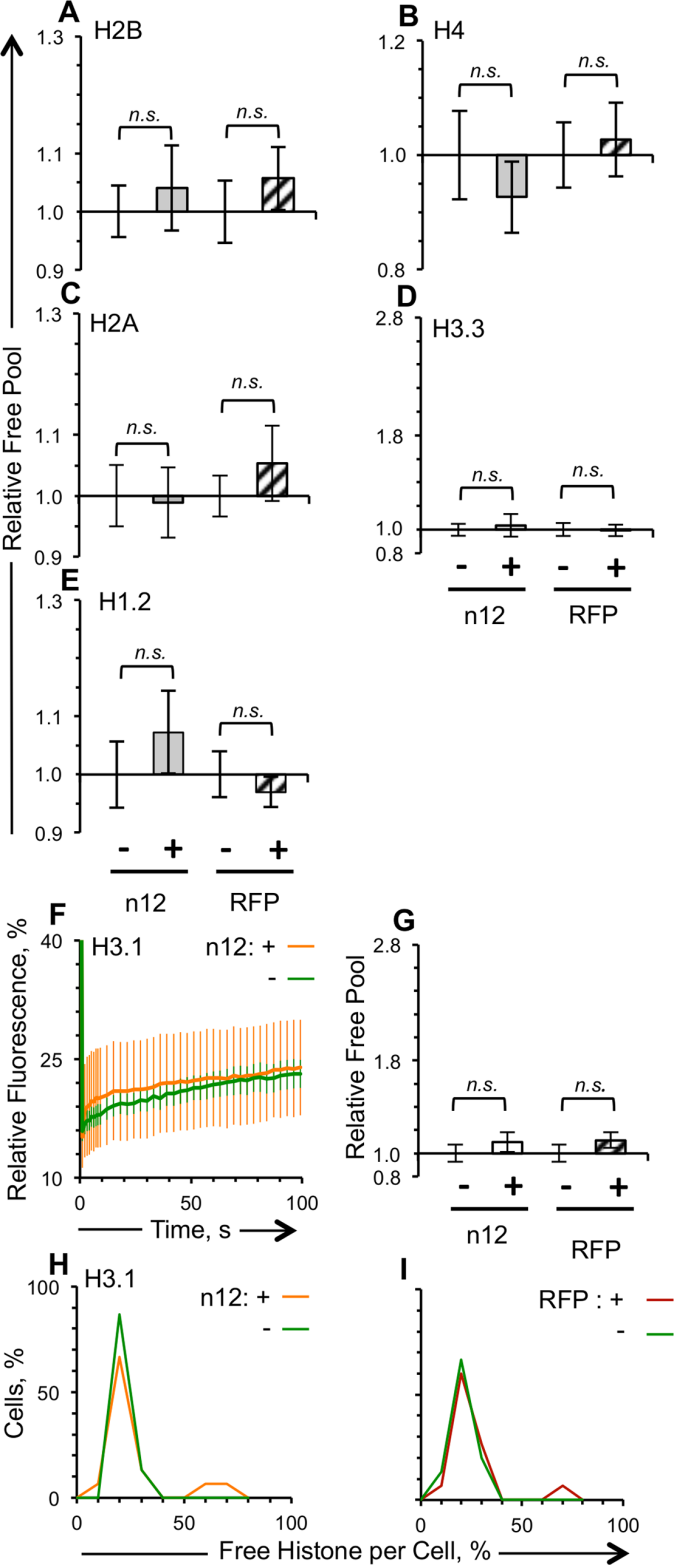
The dynamics of no histone is altered in cells transiently expressing the truncated, non-functional, ICP4 mutant n12. Vero cells were co-transfected with plasmids expressing GFP-histones and RFP-n12 or RFP such that approximately half of the cells expressing detectable levels of GFP also express detectable levels of RFP-n12. A-E), G) Bar graphs showing average levels of free GFP-H1.2, -H2A, -H2B, -H3.3, -H4, and -H3.1 in cells expressing detectable levels of RFP-n12 or RFP relative to those in cells expressing undetectable levels of RFP-n12 or RFP, respectively. F) Average fluorescence recovery curves for GFP-H3.1 in cells expressing detectable (orange line) or undetectable (green line) levels of RFP-n12. H) Distribution curve of the free pool of GFP-H3.1 in cells expressing detectable (orange line) or undetectable (green line) levels of RFP-n12. I) Distribution curve of the free pool of GFP-H3.1 in cells expressing detectable (dark red line) or undetectable (green line) levels of RFP. Error bars, SEM. **, P < 0.01; *, P < 0.05; *n*.*s*., not significant. n ≥ 15 cells from at least 3 independent experiments.

HSV-1 n12 encodes only the amino-terminal 251 amino acid residues of ICP4. This mutant is unable to activate early or late gene expression [41]. The HSV-1 n12 mutant virus barely enhanced the dynamics of any histone in Vero or U2OS cells (Figs 1 and 2). The mutant form of ICP4 was therefore not expected to alter histone dynamics. To test this model, a plasmid encoding the n12 form of ICP4 fused in frame with red fluorescent protein was constructed (RFP-n12). Mobilization of core and linker histones was analyzed in cells expressing RFP-n12. The dynamics of no histones were altered in Vero cells expressing detectable or undetectable levels of RFP-n12 (Fig 8). Free pools, fast and slow exchange, or T_50_ of no histone were affected by expression of RFP-n12. GFP-H3.1 had the expected punctuated localization in cells expressing detectable levels of RFP-n12, or of RFP (S1 Fig).

### Histone dynamics increase preferentially within HSV-1 replication compartments

**Fig 9:**
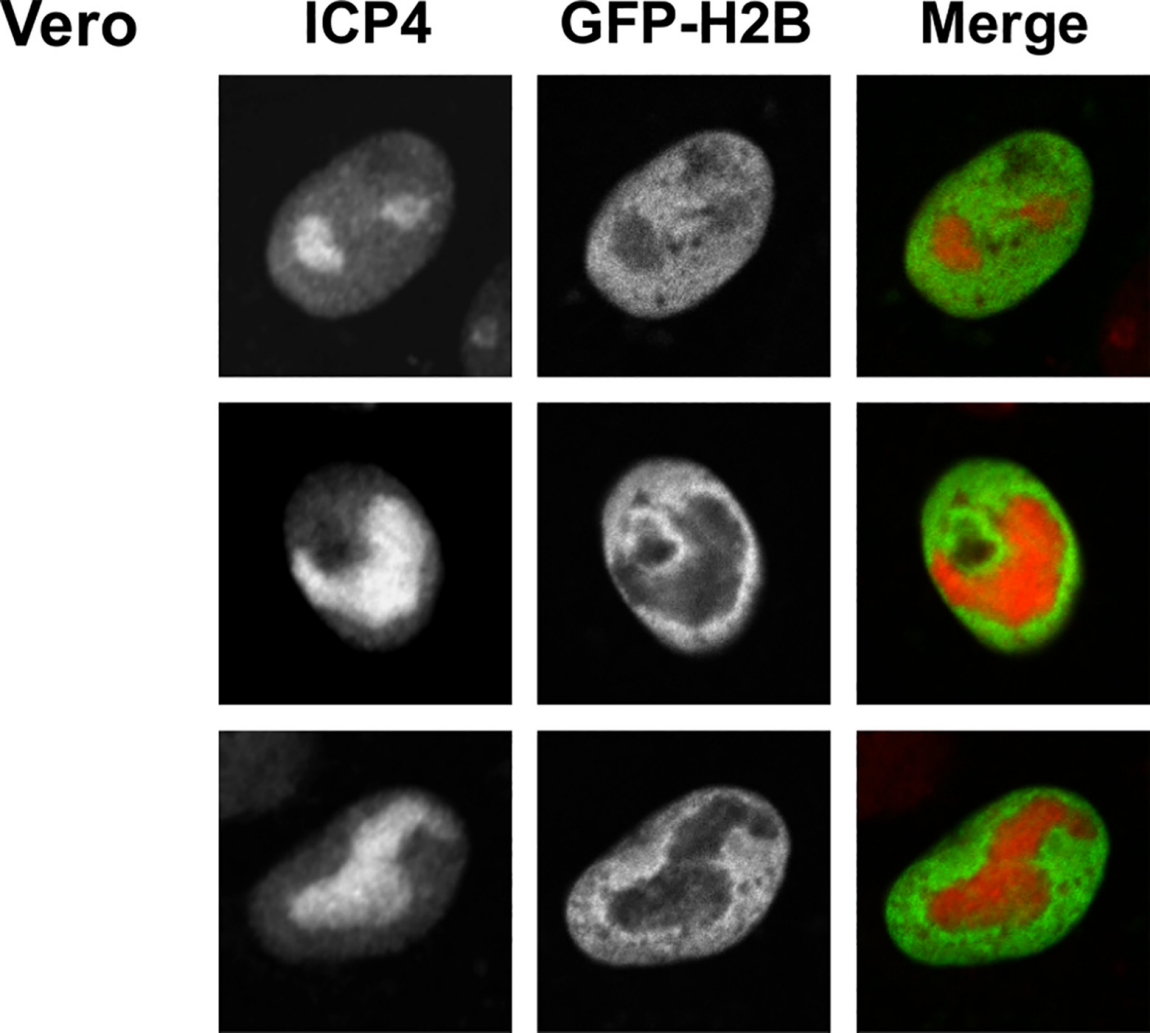
The majority of ICP4 localizes in the replication compartments with a small pool of histones. Digital fluorescent micrographs showing Vero cells expressing GFP-H2B, infected with 30 PFU of HSV-1 strain KOS, and stained with anti-ICP4 antibodies. Cells were fixed at 7 hpi and immunostained for ICP4. Single channel and merged images are shown. Note the presence of a small pool of GFP-H2B in the replication compartments, similar localization has already been reported for GFP-H1.2, -H2B, -H3.1, and -H3.3 [38–40].

HSV-1 DNA and ICP4 localize to the HSV-1 replication compartments, where they also co-localize with a small pool of histones (Fig 9). There was less fluorescence in the replication compartments than in the cellular chromatin (Fig 9), which may indicate fewer histones in the replication compartments or that the histones within the replication compartments are more dynamic and spend less time in them than in the cellular chromatin. A fluorescent micrograph cannot distinguish between 80% fewer histones or the same amount of histones having an 80% shorter residency time in the replication compartments.

**Fig 10:**
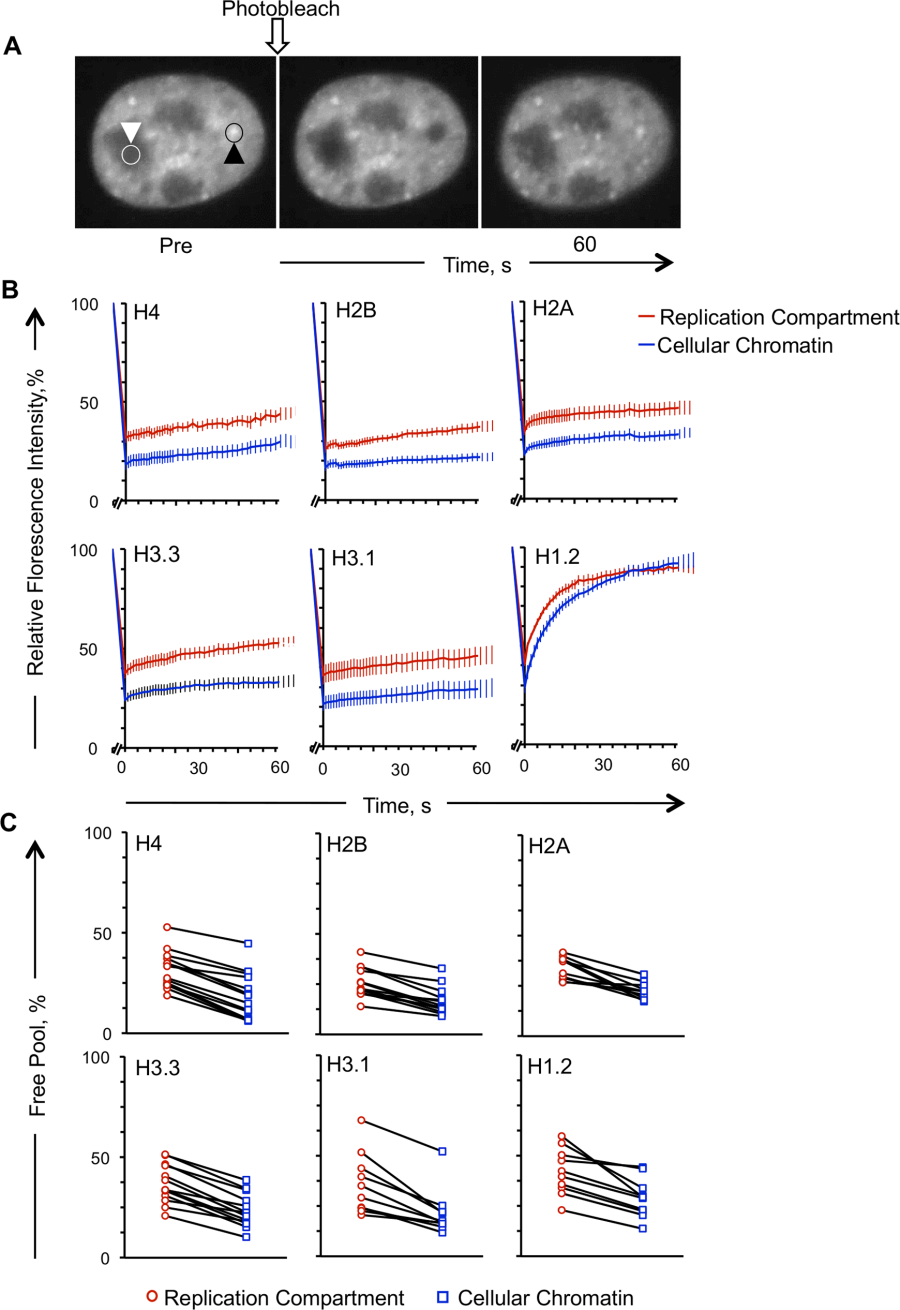
Fluorescence recovery of GFP-tagged histones in HSV-1 replication compartments or cellular chromatin. A) Fluorescence micrographs of the nucleus of an HSV-1 infected Vero cell expressing GFP-H1.2 and undergoing FRAP at 7 to 8 hpi. Left and middle micrographs, immediately prior (pre) or after photobleaching (T = 0), respectively; right micrograph, 60 seconds after photobleaching. White downward arrowhead and white circle, replication compartment region to be photobleached; black upward arrowhead and black circle, cellular chromatin region to be photobleached. Note the presence of a small pool of H1.2 in the replication compartments. B) Line graphs presenting the average ± SEM fluorescence recovery curves of histones GFP-H3.1 (n = 10), GFP-H3.3 (n = 13), GFP-H4 (n = 14), GFP-H2A (n = 10), GFP-H2B (n = 13), and GFP-H1.2 (n = 10) in the replication compartments or cellular chromatin at 7 to 8 h of infection with HSV-1, strain KOS. C) Dot plot presenting the free pools in the replication compartments or the cellular chromatin in each individual cell. Solid lines, same cells.

We therefore characterized next the histone dynamics in the replication compartments and the cellular chromatin of the same cell (Fig 10A). The free pools of core histones H2A, -H2B, -H3.1, -H3.3, and -H4, and that of linker histone GFP-H1.2, all increased preferentially within the HSV-1 replication compartments (Fig 10). GFP-H4 and -H3.1 had the largest average relative free pools in the replication compartments, 73 or 67% greater than those in the cellular chromatin, respectively (Fig 10B and 10C; p<0.01). The free pools of GFP-H2A, -H2B, and -H3.3 were 50%-56% larger in the replication compartments than in the cellular chromatin, whereas that of linker histone GFP-H1.2 had the smallest difference, 41% larger in the replication compartments than in the cellular chromatin (Fig 10B and 10C; p<0.01). The free pools were consistently higher in the replication compartments than in the cellular chromatin in all cells (Fig 10C).

The average slow exchange rates of H3.3 or H2B were 67 or 128% faster (P<0.01), respectively, in the replication compartments than in the cellular chromatin, whereas those of other histones were not statistically different.

## Discussion

It has recently been largely agreed that HSV-1 genomes are chromatinized during lytic infections [46–53], albeit the viral chromatin is far more dynamic than the cellular one [17]. Cellular chromatin containing transcribed genes is more dynamic than that containing silenced genes [21, 54–56]. The dynamics of the viral chromatin are therefore consistent with the high rate of transcription of the viral genomes during lytic infection.

A balance between cellular and viral effects may determine the unusual dynamics of HSV-1 chromatin. The assembly of nucleosomes with HSV-1 DNA may be a cellular response to inhibit HSV-1 transcription by assembling the viral genome in silenced chromatin. To counteract such silencing, HSV-1 would have evolved proteins to destabilize nucleosomes or to mobilize them away from its genome. Either mechanism would result in increased access by RNA polymerase II to the viral DNA, activating transcription. These HSV-1 proteins would thus be expected to act as transcription activators, without actually binding to specific promoter sequences.

ICP4 is one of the three HSV-1 transcription activators, and the only one required for HSV-1 replication. Though it binds to specific DNA sequences to inhibit transcription, it does not do likewise to activate it [13, 14]. Its mechanism of transcription activation remains only partially understood. Here we show that ICP4 is both necessary and sufficient to increase histone dynamics. Consistently with these findings, HSV-1 genomes are less accessible to nuclease digestion in the absence of IE proteins [57], and H3 association with HSV-1 DNA increases in the absence of functional ICP4 [58] (the changes were not statistically significant, perhaps due to the variability in the degree of the increased association for the ICP4 mutant virus).

We selected the histones to be evaluated based on the following criteria. H1.2 is expressed in all cell types that HSV-1 infects, and is mobilized the most of all linker histones [38]. H4 and H2B have no variants, and therefore represent the two core histone dimers, whereas H3 and H2A have several variants. H3.1 and H3.3 bind to HSV-1 genomes, via DNA-replication dependent or independent mechanisms, respectively [45], and their dynamics are differentially affected in HSV-1 infected cells [40]. Canonical H2A is the most prevalent H2A in nucleosomes, and no H2A variant has yet been reported to interact with HSV-1 chromatin. We analyzed histone dynamics at 4 or 7 hpi, and never beyond 8 hpi. At later times, chromatin shearing or marginalization [59] are likely to indirectly affect histone dynamics.

The HSV-1 n212 ICP0 mutant induced increases in the free pools of all histones except H4 in U2OS cells larger than those induced by the wild type virus, suggesting that ICP0 may induce the degradation of the histones in the free pools. Nonetheless, the HSV-1 mutant encoding a truncated non-functional ICP4 n12 was the most defective. This mutant either failed to enhance histone dynamics (in U2OS cells) or only enhanced them to a basal level (in Vero cells), even though it overexpresses all other IE proteins. ICP4 may therefore modulate histone dynamics by itself. Alternatively, ICP4 could indirectly affect histone dynamics through any E protein, as the expression of all E proteins requires ICP4 (DNA replication or L proteins are not required [38–40]). To test these possibilities, we constructed plasmids expressing full length or truncated forms of ICP4 fused in frame with RFP. The dynamics of all core histones except H2A increased in cells transiently expressing ICP4 but not in cells expressing the non-functional truncated n12 mutant form of ICP4.

H3.3 is initially assembled in nucleosomes with HSV-1 genomes, whereas H3.1 starts to be assembled in HSV-1 nucleosomes concomitantly with HSV-1 DNA replication [45]. Nucleosomes containing H3.3 are more dynamic than those containing H3.1 [30]. Therefore, H3.1-containing nucleosomes would be expected to be less prone to support transcription than those containing H3.3. The free pools of GFP-H3.3 increased by only 15 or 22% in Vero or U2OS cells expressing ICP4, whereas those of GFP-H3.1 increased by 248 or 40% in Vero or U2OS cells, respectively. ICP4 may thus preferentially prevent the assembly of HSV-1 genomes in the more stable H3.1 containing nucleosomes.

The free pool or slow exchange rate of GFP-H2B increased by 12% or 57%, respectively, in cells expressing ICP4. H2B forms dimers with canonical H2A or any one of its many variants. No H2A variant has yet been reported to bind to HSV-1 genomes. H2A was therefore expected to be mobilized in cells expressing ICP4. Surprisingly, it was not. It is thus most likely some other H2A variants are targeted by ICP4. Whereas H2A and H2A.X associate with both transcribed and silenced genes, for example, macroH2A preferentially associates with silenced ones [60]. Nucleosomes containing macroH2A are less dynamic than those containing canonical H2A [61, 62]. Like its differential effects on H3.1 and H3.3, ICP4 could also preferentially mobilize particular H2A variants such as macroH2A away from HSV-1 genomes.

If ICP4 itself enhanced histone dynamics, then one would expect histone dynamics to increase preferentially in the replication compartments, where ICP4 accumulates. Indeed, we found that the dynamics of all histones were faster in the replication compartments than in the cellular chromatin of the same nuclei. Though the free pools of all histones were greater in replication compartments, the slow exchange rates of only GFP-H2B and GFP-H3.3 were significantly greater, and that of GFP-H2B nearly twice as much as that of GFP-H3.3. Consistently, the slow exchange rate of only GFP-H2B was also significantly greater in cells expressing detectable levels of ICP4. HSV-1, and ICP4 in particular, may preferentially affect the less dynamic nucleosomes, which affect the slow exchange rate the most, over the more dynamic ones.

All herpesviruses appear to encode proteins that regulate chromatin dynamics. These proteins are either tegument proteins, and therefore introduced into the cell with the infected virions, or expressed immediately upon nuclear entry of the viral genome. Either way, they are all available to remodel chromatin before the activation of generalized viral gene expression. The genomes of human cytomegalovirus (HCMV) are in much less dynamic chromatin in the absence of immediate early protein 1, for example, and the Epstein-Barr virus (EBV) major tegument protein BNRF1 binds to the H3.3 chaperone Daxx, which physiologically assembles silencing H3.3-containing nucleosomes in telomeres, thus preventing silencing H3.3 incorporation in EBV chromatin [63, 64]. Nonetheless, the genomes of beta- or gamma- herpesviruses are assembled in far less dynamic chromatin than those of the alpha-herpesviruses. The genomes of HCMV and EBV are less accessible to MCN digestion than those of HSV-1 [63, 65–67], which is consistent with them being assembled in less dynamic chromatin. ChIP also co-immunoprecipitates relatively more EBV or HCMV than HSV-1 DNA, also consistent with the EBV and HCMV chromatin being less dynamic than that of HSV-1 [68–70]. Nucleosomes are also more uniformly assembled with EBV or HCMV than HSV-1 genomes [70–72], again consistent with less dynamic chromatin for the former. Alpha-herpesviruses also have much shorter replication cycles (~18 hours for HSV-1) than beta- or gamma- herpesviruses (~3 days for HCMV, ~4–5 days for EBV). ICP4 is conserved only among all alpha-herpesviruses, and not in beta- or gamma- herpesviruses. It is tempting to speculate that ICP4 may induce the particular dynamics of the alpha-herpesvirus chromatin, which would in turn result in the increased rate of transcription and consequently shorter replication cycle.

HSV-1 genes are transcribed by the cellular RNA polymerase II complex, which is enriched on HSV-1 genes while depleted from cellular genes in lytic infections [73, 74]. Nucleosomes impair accessibility of the RNA polymerase II complex to promoters DNA [21, 54–56], and the HSV-1 chromatin is far more dynamic and accessible than the cellular one [17]. ICP4 may maintain the HSV-1 genomes in this dynamic and highly accessible chromatin, resulting in the RNA polymerase II complexes being sequestered away from the cellular genome and to the HSV-1 genomes [74], thus leading to the activation of HSV-1 transcription and inhibition of cellular transcription.

In summary, we show that the HSV-1 transcription activator ICP4 is sufficient and necessary to enhance histone dynamics. ICP4 preferentially affects the silencing histone H3.1 over the activating variant H3.3, and it does not affect canonical H2A. ICP4 may therefore target silencing histones, preventing them from assembling silencing nucleosomes with HSV-1 genomes, or mobilizing them away from HSV-1 nucleosomes, to activate HSV-1 gene transcription. This mobilization may function to counteract a cellular defense mechanism against dsDNA viruses involving chromatin silencing.

## Materials and Methods

### Cells and viruses

African green monkey Vero cells and their HSV-2 ICP4 expressing derivative n-33 cell line (a generous gift from the late Dr. P. Schaffer; [44]) were maintained in Dulbecco’s modified minimum Eagle’s medium (DMEM) supplemented with 5% fetal bovine serum (FBS) at 37°C in 5% CO_2_. Human osteocarcinoma U2OS cells (a generous gift from Dr. J. Smiley, University of Alberta) were maintained in Dulbecco’s modified minimum Eagle’s medium (DMEM) supplemented with 10% FBS at 37°C in 5% CO_2_.

Wild-type HSV-1 strain KOS and mutant strain n12 (generous gifts from the late Dr. P. Schaffer) are described [75, 76]. KOS viral stocks were prepared and titres were determined by standard plaque assay as described [38–40]. Preparation of n12 viral stocks and determination of n12 titres was as for KOS except that n-33 cells were used instead of Vero cells. Parallel n12 titrations of Vero cells ensured that the genetic defect in ICP4 was not rescued through recombination with HSV-2 ICP4 during viral stock preparation.

### Plasmids

The green fluorescent protein (GFP)-histone fusion plasmids were described previously [38,39, 40, 42]. pEGFP-H3.3 was a generous gift from Dr. John Th’ng (Northern Ontario School of Medicine). The amino-terminal 2300 base pairs of ICP4 were amplified from HSV-1 DNA using primers 5’ AGA TCT CCG GAG GAT CGC CCC GCA TCG and 5’ CGT CCG AGC CGG GGG CGT CCG and the carboxy-terminal 1800 base pairs using primers 5’ CGG CGG CCC GCG ACC CCC and 5’ TCT AGA TCA CAA GCG CCC CGC CCC. The ICP4 PCR fragments were digested with SapI, and the amino- and carboxy- fragments were ligated with T4 ligase (Invitrogen). Full length ICP4 was then cloned into the BglII and XbaI sites of the pmCherry-C1 vector (Clontech).

### Transfections and infections

U2OS, Vero, and n-33 cells were transfected with Lipofectamine 2000, seeded onto coverslips, and infected for FRAP basically as described [38–40].

For co-transfections, 2.2–2.4x10^5^ Vero or U2OS cells were transfected with 14 or 4 μl, respectively, of Lipofectamine 2000 reagent (Invitrogen), 0.2 or 1 μg, respectively, of GFP-histone plasmid and 1.8 or 1 μg, respectively, of RFP-ICP4, RFP-n12 or RFP-expressing plasmids. Following 6.5 h incubation with the transfection mix, 1 ml of 37°C DMEM medium supplemented with 10% FBS was added to the cells. Cells were seeded onto coverslips at least 4 h later. Cells were incubated at 37°C for at least 12 (GFP-H2A, -H2B, -H3.3, or -H1.2) or 24 (GFP-H3.1 or -H4) h after transfection before subsequent infection. Transfected cells were seeded onto coverslips and infected for FRAP as described [38–40].

### Fluorescence recovery after photobleaching (FRAP)

Histone mobilization was evaluated by FRAP 24–48 h after transfection essentially as described [38, 39]. A 1.5 μm-wide region spanning the nucleus was photobleached, with 30 to 45 iterations at 95% intensity. Fifteen or more cells from at least three independent experiments were evaluated per treatment. Statistical significance was tested using one-tailed Student’s T test (for two-way comparisons) or ANOVA (for multiple comparisons).

### Immunofluorescence

ICP4 localization was visualized by immunofluorescence as described [38].

### FRAP in viral replication compartments versus cellular chromatin

Equal volumes of replication compartments or cellular chromatin in the same infected cells (Fig 10A) were photobleached at 7 hours post infection (hpi) with 10 PFU/cell of HSV-1, strain KOS. The fluorescence recovery within the photobleached regions was monitored once per second for the first 20 seconds and once every two seconds for the next 40 seconds. Background fluorescence was subtracted and fluorescence was normalized to that of the entire nucleus. Fluorescence at any given time is expressed as a percentage of the normalized fluorescence intensity within the same photobleached region (replication compartment or cellular chromatin) immediately before photobleaching.

## Supporting Information

S1 Fig RFP or RFP-n12 do not alter the nuclear distribution of H3.1. Representative images of fluorescent nuclei expressing GFP-H3.1 and detectable or undetectable levels of RFP or RFP-n12, immediately prior to (T = 0) or after (T = 1) photobleaching, or 200 seconds later.
(PDF)
