# Supplementary material for: An Essential Viral Transcription Activator Modulates Chromatin Dynamics
Source: PLoS Pathog. 2016 Aug 30;12(8):e1005842. doi: 10.1371/journal.ppat.1005842 (PMC5004865; doi:10.1371/journal.ppat.1005842)
Supplement: S1 Fig — Representative images of fluorescent nuclei expressing GFP-H3.1 and detectable or undetectable levels of RFP or RFP-n12, immediately prior to (T = 0) or after (T = 1) photobleaching, or 200 seconds later. (PDF) [file ppat.1005842.s001.pdf]

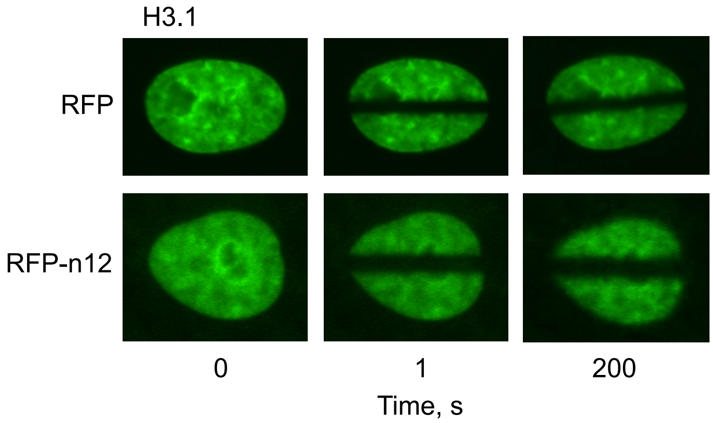

S1 Fig. RFP or RFP-n12 do not alter the nuclear distribution of H3.1.
Representative images of fluorescent nuclei expressing GFP-H3.1 and detectable or undetectable levels of RFP or RFP-n12, immediately prior to (T = 0) or after (T = 1) photobleaching, or 200 secs later.
